# Analysis of High-Throughput Sequencing and Annotation Strategies for Phage Genomes

**DOI:** 10.1371/journal.pone.0009083

**Published:** 2010-02-05

**Authors:** Matthew R. Henn, Matthew B. Sullivan, Nicole Stange-Thomann, Marcia S. Osburne, Aaron M. Berlin, Libusha Kelly, Chandri Yandava, Chinnappa Kodira, Qiandong Zeng, Michael Weiand, Todd Sparrow, Sakina Saif, Georgia Giannoukos, Sarah K. Young, Chad Nusbaum, Bruce W. Birren, Sallie W. Chisholm

**Affiliations:** 1 The Broad Institute of MIT and Harvard, Cambridge, Massachusetts, United States of America; 2 Department of Civil and Environmental Engineering, Massachusetts Institute of Technology, Cambridge, Massachusetts, United States of America; Universidad Miguel Hernandez, Spain

## Abstract

**Background:**

Bacterial viruses (phages) play a critical role in shaping microbial populations as they influence both host mortality and horizontal gene transfer. As such, they have a significant impact on local and global ecosystem function and human health. Despite their importance, little is known about the genomic diversity harbored in phages, as methods to capture complete phage genomes have been hampered by the lack of knowledge about the target genomes, and difficulties in generating sufficient quantities of genomic DNA for sequencing. Of the approximately 550 phage genomes currently available in the public domain, fewer than 5% are marine phage.

**Methodology/Principal Findings:**

To advance the study of phage biology through comparative genomic approaches we used marine cyanophage as a model system. We compared DNA preparation methodologies (DNA extraction directly from either phage lysates or CsCl purified phage particles), and sequencing strategies that utilize either Sanger sequencing of a linker amplification shotgun library (LASL) or of a whole genome shotgun library (WGSL), or 454 pyrosequencing methods. We demonstrate that genomic DNA sample preparation directly from a phage lysate, combined with 454 pyrosequencing, is best suited for phage genome sequencing at scale, as this method is capable of capturing complete continuous genomes with high accuracy. In addition, we describe an automated annotation informatics pipeline that delivers high-quality annotation and yields few false positives and negatives in ORF calling.

**Conclusions/Significance:**

These DNA preparation, sequencing and annotation strategies enable a high-throughput approach to the burgeoning field of phage genomics.

## Introduction

The sheer abundance and ecological importance of phage in most environments, coupled with limited knowledge of their genetic makeup, demands establishing genomic methods that can be applied at scale and implemented to decipher the genetic frameworks that drive phage biology. To date, obtaining a complete genome sequence remains one of the most efficient ways to gain insight into the biology of an organism, especially for a microbe whose biology may be difficult to study in its natural environment or in the laboratory. Recent rapid advances in sequencing technologies and sample preparation methods are changing the landscape of what is possible regarding complete genome sequencing of organisms such as phages, providing a window into understanding how these important organisms modulate microbial communities, and by extension, impact ecosystem function and human health.

The importance of phages in marine systems cannot be overstated. With concentrations exceeding 10 million per milliliter of seawater [1], they are likely the most abundant forms of life in the Earth's oceans, harboring a tremendous amount of genetic diversity [2]. These phages play a role in both horizontal gene transfer and host mortality of the microbial populations that are responsible for the biogeochemical processes that run the planet [3], [4], thus shaping the ecology and evolution of both over evolutionary time. However, we have only barely begun to understand the genomic repertoire of these important genetic vectors [5], [6].

A tiny sampling of cultured marine phage genomes and community DNA metagenomic sequencing has led to the following broad observations. First, the few cultured isolates appear to resemble known phage types, such as the T7-like [7]–[10], P2-like [11], [12] and T4-like [9], [13], [14] phages, suggesting phage evolution might occur by incremental modulations of a common organizational pattern or chassis. Random ‘metagenomic’ sequencing of amplified viral DNA from microbial communities [15], [16], [2] and unamplified cellular DNA [17], [18] supports the prevalence of these types but also suggests others exist in the wild. Second, marine phages appear to have acquired and altered critical host metabolic genes, presumably needed to enhance phage fitness. For example, known cyanophage genomes encode a suite of proteins involved in photosynthesis, including the core reaction center proteins, D1 and D2 [19]–[22]. These genes are expressed during infection [23], [24], presumably to ensure sufficient photosynthetic capacity of the host for the duration of the infection, likely necessitated in part because the D1 protein is prone to damage and rapid turnover. The phage versions of ‘host’ photosynthesis genes may be subject to selective pressures different from those of the host, thus allowing new genetic variation to be generated and possibly to find its way back into the host [25], [22]. Thus cyanophages may potentially drive the evolution of photosystems on a global scale [26], and there are undoubtedly other metabolic pathways of biogeochemical importance that they also influence. Finally, marine RNA viruses have recently been discovered and described, though their hosts are not yet known and their genomic diversity has hardly been described [27].

Despite their clear global significance, genome sequencing of marine and other phages has been limited–in part because of technical obstacles. First, culturing most marine host cells for phage infection has been a major challenge, only recently yielding to new high-throughput culturing efforts [28]. In addition, obtaining sufficient phage genomic DNA (gDNA) for sequencing has been difficult, in part due to slow growth rates of most marine microbes, and cumbersome growth and purification procedures required to obtain sufficient phage particles. Further, methods for sequencing phage genomes at scale cannot require *a priori* knowledge of the genome, since traditional primer-based approaches require primer design and are too labor and cost intensive to be applied at scale.

Here we delineate a streamlined genomic DNA sample preparation method directly from crude phage lysates that can be used with any sequencing strategy, and compare the results of several sequencing approaches. The sequencing strategies evaluated include an optimized linker amplified shotgun library (LASL), a whole genome shotgun library (WGSL), and 454 pyrosequencing. They were compared for their ability to deliver high-quality, accurate, complete genome assemblies. The results demonstrate that, although LASL amplification was minimally biased, the cloning biases seen with both the LASL and WGSL methods resulted in incomplete genomes which require costly and time-consuming finishing reactions to close the gaps. In contrast, the 454 sequencing approach both eliminated the cloning bias issue and proved sufficient for obtaining robust *de novo* genome assemblies. Lastly, we describe a high-throughput automated annotation pipeline for the calling of genes from sequenced phage genomes.

## Results and Discussion

### DNA Template Purification

Phage DNA used for sequencing has traditionally been extracted from highly purified phage particles isolated via cesium chloride (CsCl) density gradients. While this process most cleanly separates phage particles from non-phage DNA and particulates in the lysates, the method is cumbersome and time-consuming, and in the case of ocean cyanophages, often results in high particle loss and low DNA yield (M. Sullivan, unpublished results). Further, reduced sequencing costs and the increased ability to filter out non-phage DNA sequence reads at the genome assembly stage now obviate the need for gDNA of such high purity. This, coupled with the need for higher-throughput marine phage DNA template preparation pipelines, led us to devise and optimize a process, based on a protocol originally designed for coliphage lambda (Promega, Madison WI), for purifying DNA from relatively small volumes (100–200 ml) of crude phage lysates containing approximately 10^8^ phage particles per milliliter (see Methods). Starting with an equivalent number of phage particles in a crude lysate, DNA yields from the lysate prep method (0.1–1.0 mg of DNA from a 100-ml lysate) were at least ∼2–30X higher than those obtained from PEG-concentrated-CsCl purified phages (see Methods).

To further optimize and streamline the lysate prep method, we explored the possibility of eliminating a nuclease treatment step that was included in the original protocol in order to reduce contaminating host DNA. To this end, gDNA from cyanomyovirus S-SM1 was prepared in triplicate with and without nuclease treatment. Treatment with nucleases did not impact the sequencing output. When assayed using 454 sequencing, all replicates of the S-SM1 genome resulted in single contig assemblies comprised predominantly of high quality bases (Table 1). Nuclease treatment had no impact on phage genome assembly length, which in most cases was within 2 bases of the reference assembly. The fraction of junk/contaminating reads (i.e. non-phage gDNA typically of host origin) was not statistically different among nuclease treatments when measured as the fraction of singleton reads (paired t-test, p = 1.00) or fraction of unassembled reads (paired t-test; p = 0.36). The fraction of singleton reads ranged from 0.07 to 0.84, and the fraction of unassembled reads ranged from 0.16 to 0.24.

**Table 1:** Impact of lysate nuclease treatment on 454 assembly.

Library ID	Nuclease +/−	No. of Phage Contigs	Total Contig Length (bp)	Largest Contig Length (bp)*	Fraction of bases >Q40
519	+	1	175,091	174,078	99.7%
520	+	1	174,060	174,060	99.9%
521	+	1	175,170	174,079	99.9%
522	−	1	174,079	174,079	100.0%
523	−	1	174,081	174,081	99.9%
524	−	1	174,070	174,070	99.1%

*Genome size of P-SM1 is 174,079 bp.

These successful genome assemblies, combined with the decreased template preparation time (2 hrs for lysate preps vs 8 hrs for CsCl preps), suggest that purification of phage DNA directly from crude lysates is more suitable for high-throughput genomic work.

### Sanger Sequencing Strategy Using Linker Amplification and Whole Genome Shotgun Libraries

Previously, three amplification methods have emerged to deal with the problem of low gDNA yields from marine phage preparations or uncultivated viral-fraction seawater samples: randomly amplified shotgun libraries (RASLs) [29], linker amplification shotgun libraries (LASLs) [15], [30], and phi29-based whole genome amplification [31]. Because the latter method is now known to be prone to biases in final representation of template material, likely due to stochastic initial interactions [32], we did not explore this method further. However, both the RASL and LASL methods promise access to low amounts of gDNA (with no prior knowledge of the genome sequence), could convert modified phage gDNA bases into non-modified bases, and are thought to yield a relatively non-biased amplification [29]. Thus we chose to further optimize the LASL strategy, and then evaluate possible amplification and cloning biases inherent in the LASL process. A LASL approach was selected over a RASL strategy as in the RASL protocol genomic DNA is sheared by restriction digest a process likely to have more bias than other shearing methods.

**Figure 1:**
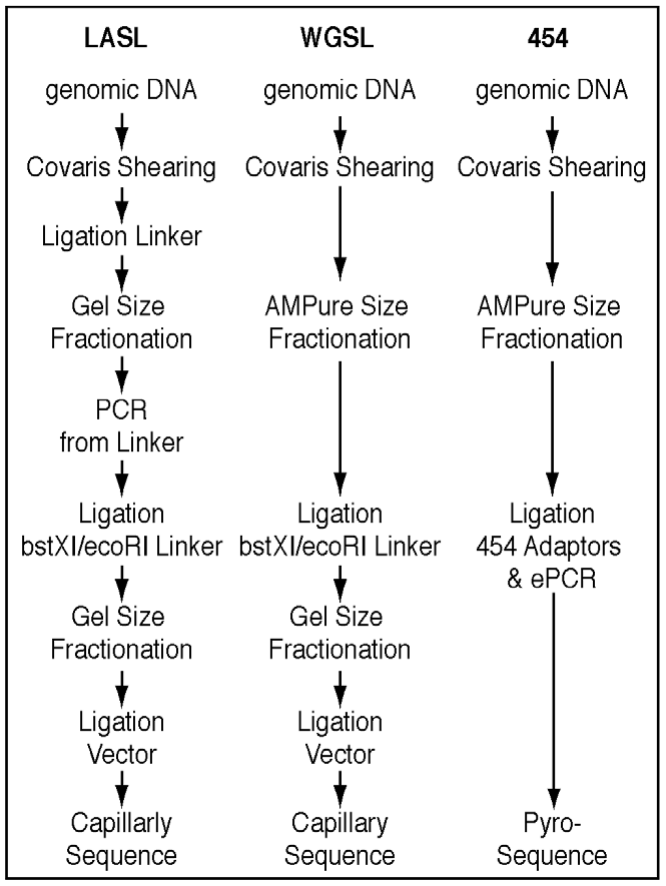
Overview of Linker Amplified Shotgun Library (LASL), Whole Genome Shotgun (WGSL), and 454 library construction strategies.

We first optimized the LASL method (Figure 1) by reducing template contamination during shearing, and taking steps to minimize cloning biases, particularly for AT-rich templates. Standard LASL-prep shearing methods can lead to contamination across samples (e.g., hydroshear) or uneven shearing (e.g., enzymes). Thus, we implemented the High Frequency Adaptive Focused Acoustics (AFA) technology from Covaris Inc. (Woburn MA) to randomly shear the gDNA to the desired size range. Treatment of gDNA samples with non-contact controlled isothermal mechanical energy in an enclosed environment prevented sample loss as well as cross-contamination. Acoustic shearing of nanogram quantities of DNA was highly reproducible, and conditions were optimized for obtaining 1.2–1.5 kb DNA fragments for LASL construction. After shearing, we took steps to minimize cloning biases, particularly for AT-rich templates, as follows: (1) eliminating steps requiring elevated temperatures or high salt concentration to minimize DNA loss through denaturation, (2) adding a second size fractionation step after addition of the BstXI/EcoRI adaptor to tighten the insert size range and to remove excess linker, and (3) ligating the DNA into a low-copy vector.

**Figure 2:**
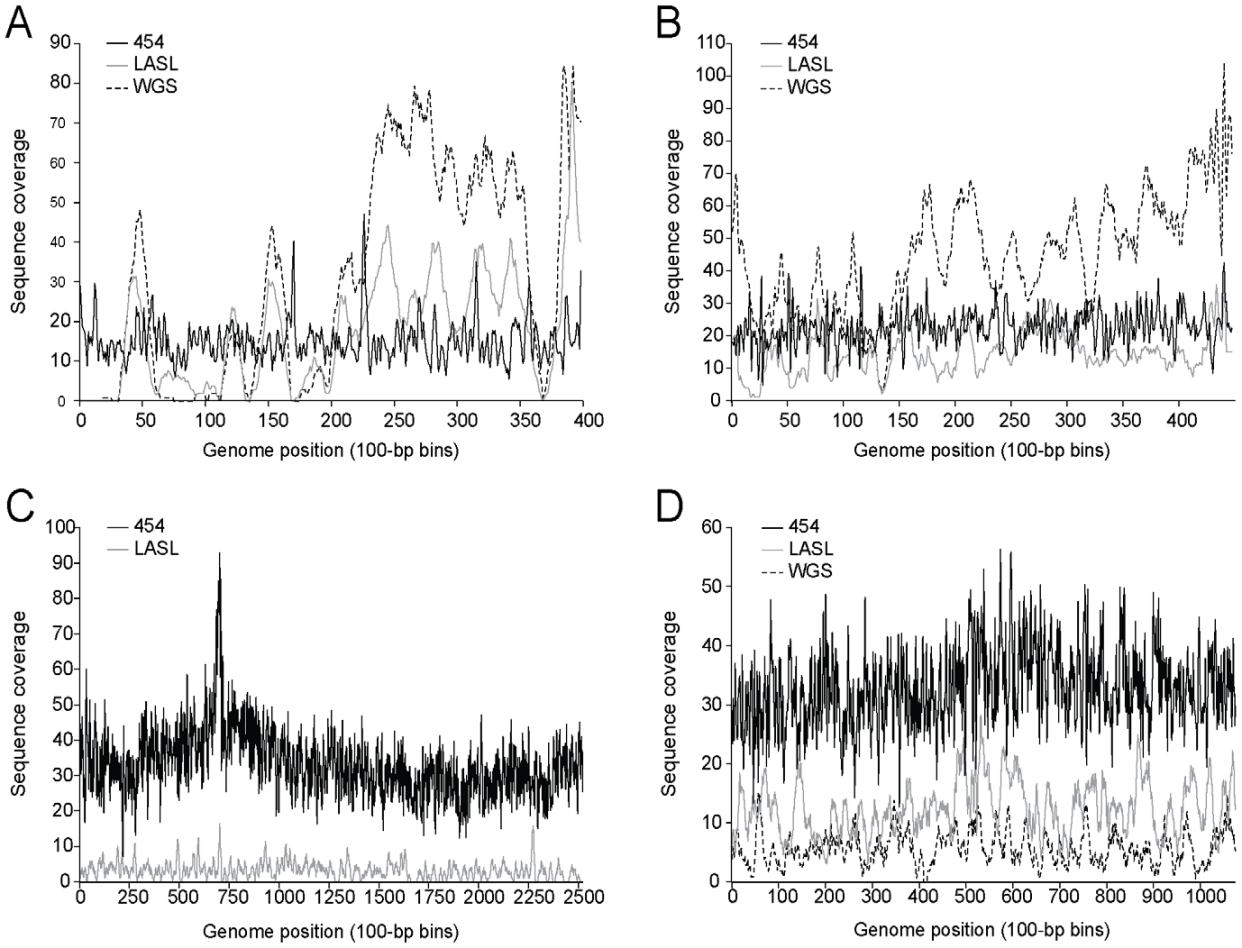
Comparison of sequence coverage across genomes sequenced using the 454, LASL, or WGSL approaches. Coverage plots for T7 (A), P-SSP7 (B), P-SSM2 (C), and P-SS2 are shown. Sequence coverage is binned by 100 nt windows.

To initially evaluate possible amplification and/or cloning biases, we compared the distribution of random Sanger sequenced shotgun reads from 3 phage DNA templates (coliphage T7, and cyanophages P-SS2, P-SSP7) prepared using both (a) the optimized LASL method, and (b) a standard whole genome shotgun library (WGSL) construction process. The WGSL method was suitable at input template DNA concentrations achieved with the lysate sample preparation method and WGSL has the advantage of not requiring a linker ligation or PCR step (Figure 1). For all 3 phages, the genome sequence coverage was similar using either LASL or WGSL, but both methods also showed under-representation in similar regions of the genomes (Figure 2). These results suggest that the amplification step of the LASL process was relatively unbiased, and that under-representation in both cases was likely due to cloning bias (e.g., toxic or AT-rich genes).

To confirm this analysis, we developed a qPCR assay to determine whether steps prior to cloning contributed to the observed coverage bias. Twelve regions of the P-SS2 genome that represented areas of high and low sequence coverage were interrogated (Figure S1). As we did not observe significant differences between products amplified from different regions of the genome as a result of: i) Covaris shearing, ii) steps prior to vector ligation in the LASL method, or iii) steps prior to vector ligation in the WGSL protocol (Figure 1), it is likely that all regions of the genome were initially present prior to cloning and that the uneven assembly was instead a result of cloning bias.

**Figure 3:**
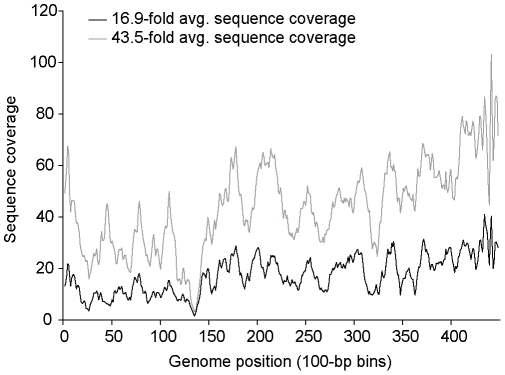
Bias at low and high sequence coverage of P-SSP7 genome sequenced using the WGSL approach. Sequence coverage is binned by 100 nt windows.

While the sequencing and assembly of the reference phages were comparable using either the LASL or WGSL method (Table 2), the LASL approach on average required considerably less DNA (as little as 0.5 ng as compared10–50 ng for the WSGL approach). WGSL library construction protocol was also attempted on a single CsCl sample prep using only 10 ng of input DNA, and yielded similar results (data not shown). Contaminating DNA (i.e., reads with significant BLAST hits to non-phage entries in Genbank's non-redundant database) was minimally present in both the LASL (1.20%±0.03%) and WGSL (0.26%±0.00%) preps. Again, however, neither method captured complete genomes due to the highly biased sequence coverage across the target genomes. This was empirically determined by increasing the sequence coverage 2.5-fold (to 43.5-fold total coverage) on the P-SSP7 WGSL library; this reduced the number of viral-contigs in the assembly (from 7 to 4), but did not improve the coverage bias (Figure 3).

**Table 2:** LASL and WGSL assembly metrics.

Reference Phage	Reference Phage Size (bp)	Library Type	Input DNA quantity (ng)	Average Sequence Coverage	% Reference Covered	No. of Phage Contigs	Largest Contig Length (bp)	Contig N50 (bp)	Percent of bases ≥Q20
P-SS2	107,530	LASL	0.5	5.8±3.0	98.1%	7	42,908	37,263	94.0%
P-SS2	107,530	LASL	1.0	5.7±2.9	97.4%	12	39,421	31,931	91.0%
P-SS2	107,530	WGS	50.0	5.3±2.6	98.3%	22	10,683	4,457	98.0%
P-SSM2	252,401	LASL	<0.5	4.3±2.9	93.0%	49	9,729	3,849	99.2%
P-SSM2	252,401	LASL	1.0	3.1±2.3	88.6%	27	4,631	3,159	99.0%
P-SSP7	44,970	LASL	1.0	13.6±6.1	99.9%	13	14,001	4,483	99.4%
P-SSP7	44,970	WGS	400.0	16.9±7.4	100.0%	7	16,602	13,164	99.2%
T7	39,937	LASL	1.0	15.9±13.1	89.7%	6	16,857	16,857	99.6%
T7	39,937	WGS	>5000	66.3±56.5	91.8%	9	14,369	6,148	99.2%

### Pyrosequencing Strategy

To circumvent cloning bias issues, we next tested the ability of pyrosequencing technology [33] to (a) generate genome sequence from small amounts of phage DNA, and (b) allow *de novo* assembly of whole phage genomes. Unlike the LASL and WGSL methods that rely on cloning sheared gDNA into a vector, pyrosequencing involves amplification of DNA ligated to beads, and then sequencing of the amplified DNA using flow cell technology; no cloning steps are required (Figure 1). However, these methods normally require microgram quantities of gDNA for library construction. Thus, we optimized the preparation of pyrosequencing libraries for use with lower input DNA concentrations by including a post-shearing DNA concentration step (see methods). This modification enabled the generation and sequencing of libraries from input phage gDNA quantities ranging from 1 ng to 500 ng (Table 3).

**Table 3:** 454 assembly metrics.

Phage*	DNA template	Input DNA quantity (ng)	Average Aligned Sequence Coverage	No. of Contigs	No. of Phage Contigs	Largest Contig Length (bp)	Total Contig Length (bp)	Percent of the Reference Covered	Percent of bases ≥Q40	454 Platform (assembled read average length in bp)
P-SS2	CsCl purified particles	1	65.2±14.4	1	1	105,532	105,532	99.4%	99.9%	GS20 & FLX (161.5)
P-SSM2	Lysate	4	33.1±9.0	23	1	252,407	252,407	100.0%	99.5%	GS20 & FLX (239.6)
P-SSP7	CsCl purified particles	4	22.5±5.7	3	3	39,777	44,935	99.9%	99.4%	GS20 (103.6)
T7	Epicenter Biotech-nologies	500	15.3±5.2	1	1	39,778	39,778	100.0%	99.8%	GS20 & FLX (145.8)

*See Table 1 for reference genome size.

The success of a 454 sequencing strategy was evaluated by assessing the evenness of the coverage across the genome and the ability to assemble *de novo* the target genome. Comparisons of the 454 genome assemblies for cyanophages P-SS2 (GenBank Accession No. GU071090.1), P-SSM2 (GenBank Accession No. GU071092.1), P-SSP7 (GenBank Accession No. GU071093.1), and coliphage T7 (GenBank Accession No. GU071091.1) to the available reference sequences at NCBI (obtained using Sanger sequencing technology) indicated that assembly of resulting 454 read data using the Newbler software (see Methods) reliably captured complete or near-complete genomes with high accuracy (Table 3). This is in contrast to the LASL and WGSL methods. Sequence coverage across each genome was more even than the coverage obtained using the clone-based LASL and WSGL Sanger sequencing strategy (Figure 2). Further, while the 454 sequence methodology did result in regions of high and low coverage, this variation did not impact assembly, nor was there an apparent sequence context to the regions of high versus low coverage. The standard deviation of the average sequence coverage for the phage genomes using 454 was similar to that observed when sequencing prokaryotic genomes using this technology.

The assemblies for pyrosequenced genomes were highly comparable (>99.4% sequence identity) to the Sanger sequenced genome for all four phage genomes (Table S1). Most discrepancies from the published sequence were single base pair substitutions (per genome avg: 5.25, range: 2–8) or indels (per genome avg: 6.5, range: 1–15). Of the indels, 88% of these were in homopolymeric regions consisting of ≥4 bases; such regions are known to be problematic for pyrosequencing. The most problematic genome, siphovirus P-SS2, also had 2×652 bp and 1×707 bp tandem repeat regions that were collapsed by the Newbler assembler (Table S1). Had these been biologically the same piece of DNA, one would expect 3 times the average genome coverage in this region. However, these appear to be real tandem repeats because the sequence coverage across each of the three repeats did not statistically deviant from the average genome coverage (ANOVA; p = 0.157). Sequence coverage in the three repeat regions was 75-, 69-, and 68-fold coverage as compared to 65-fold across the genome (Table 3). For genome projects that may need to resolve such problematic repeat regions, utilizing assembly statistics such as coverage in combination with either paired-reads, or targeted Sanger sequencing [34], [35] could be useful.

We did not observe a clear relationship between input template DNA concentration and 454 sequence read yield or quality; evaluation of the total number of reads relative to the number expected versus input template quantity did not show a template dependent difference in the read ratio. Notably, different DNA preps may have different ratios of target DNA to contaminants such as host DNA, or 454-adaptor dimmers that may impact the number of reads that are of the intended target. Our results suggest that fluctuations in read yield and sequence quality are more likely driven by individual run performance than by input template amount.

### Effect of Exogenous DNAs on Cyanophage Genome Assemblies

The presence of non-phage template DNAs (i.e. contaminating DNAs typically of host origin) in lysate-prepared phage DNA did not prevent assembly of the target genome by the Newbler assembly software. *In silico* assembly experiments using cyanophage S-SM1 indicated that approximately 13-fold sequence coverage is required to robustly capture a complete genome of appropriate size (Table 4). BLAST analysis of the other assembled contigs indicated origins from host, phage, or unknown DNA sources.

**Table 4:** 454 assembly quality as a function of sequence coverage.

Sequence Coverage*	Total Large Contigs (>500 nt)	Total Large Contig Length	Largest Contig Length	Largest Contig Sequence Coverage	Percent of bases >Q40
8.5	11	174,742	65,299	9.24	97.9
10.2	6	175,215	84,315	10.98	98.8
11.5	3	175,483	173,969	11.61	99.1
13.2	4	176,170	174,079	13.36	99.5
16.6	10	179,923	174,080	17.06	98.9
20.5	16	184,250	174,079	21.5	98.7
30.1	100	239,442	174,079	40.47	93.5
30.8	123	254,225	174,079	43.86	92.6

*Only lower sequence coverages are shown.

Because phage isolated from environmental samples may, despite purification attempts, occasionally contain more than one phage type, we carried out *in silico* assembly experiments to determine the success of the Newbler assembly algorithm in cases when a sample may contain multiple distinct phage genomes. We found that with a fragment length (n) of at least 100 bp, up to three different genomes from a mixed sample could be successfully assembled using Newbler, regardless of the number and relative abundances of different phage types in the sample (Table 5). In general and surprisingly, increasing the sequence read length did not improve the number of complete genomes recovered (Table 5). In these experiments, successful assembly of mixed samples was not dependent on the abundance of repetitive sequences or on the GC content of the genomes (Table S2). Unfortunately, given proprieties surrounding the Newbler software it is difficult to further interrogate the cause of the above results as the intricacies of this assembly algorithm are unknown.

**Table 5:** Results of *in silico* mixed sample assembly experiments.

Input Genomes†	Fragment length	Ratio	No. of Large Contigs	Genomes assembled*
PSSM2/PSSM4/PSSP7	100	1∶1∶1	3	PSSM2/PSSM4/PSSP7
PSSM2/PSSM4/PSSP7	425	1∶1∶1	3	PSSM2/PSSM4
PSSM2/PSSM4/PSSP7/MED4-259/MED4-247	100	1∶1∶1∶1∶1	83	PSSM2/PSSM4/PSSP7
PSSM2/PSSM4/PSSP7/MED4-259/MED4-247	425	1∶1∶1∶1∶1	28	MED4-259/MED4-247/PSSM2/PSSM4
**PSSM2**/PSSM4/PSSP7	100	2∶1∶1	3	**PSSM2**/PSSM4/PSSP7
**PSSM2**/PSSM4/PSSP7	425	2∶1∶1	3	**PSSM2**/PSSM4
PSSM2/**PSSM4**/**PSSP7**	100	1∶2∶2	3	PSSM2/**PSSM4**/**PSSP7**
PSSM2/**PSSM4**/**PSSP7**	425	1∶2∶2	3	PSSM2/**PSSM4**

† Genome names in bold were represented twice as often in the mixed samples with non-uniform ratios of sequences.

*If a genome is included in the Genomes assembled column, then a large contig from the Newbler assembly output was matched to a genome of the appropriate size using MUMMER.

### Genome Annotation

Automated annotation of the reference genomes T7, P-SSP7, and P-SSM2 using the method described here (referred to hereafter as AA; see Methods) produced comparable annotations to those previously described in GenBank. The AA pipeline identified 49, 52, and 331 genes in T7, P-SSP7, and P-SSM2 respectively, as compared to 60, 53, and 329 in the reference annotation (Table 6). In general, the two annotations called the same loci, with each method missing 10 or less of the loci predicted by the other. The discrepancy in the T7 annotation results from the presence of 11 genes that overlap the same locus and are designated as hypothetical gene predictions in the GenBank annotation. Notably, these overlapping genes are known to have biological functions [36]. The majority of predicted genes for both annotations agreed with regard to gene starts and stops (Table 6), and those that did not typically differed in the selected start codon. The AA method missed only a single potential gene and hence had a very low false-negative rate, relative to the GenBank annotations. When all BLAST hits identified during the annotation process were clustered, and these clusters subsequently mapped to predicted genes, only a single cluster did not map to a predicted gene (Table 6). On average, the AA pipeline computed complete annotation of a phage genome in 10 CPU hrs; manual inspection of the gene calls that were flagged as having potential issues required approximately one day per genome. The automated annotation pipeline described here is suitable for annotating phage genomes in a high-throughput environment.

**Table 6:** Performance of phage annotation pipeline.

Genome	Genome Size (bp)	No. ORFs GenBank RefSeq	No. ORFs Annotated Broad	No. ORFs Same Start & Stop	No. ORFs Same Start Different Stop	No. ORFs Same Stop Different Start	No. ORFs in GenBank Only	No. ORFs in Broad Only	No. blastLoci Not In GenBank	No. blastLoci Not In Broad
T7	39,778	60	49	46	3	4	11*	0	0	0
P-SSP7	44,935	53	52	47	1	3	2	1	1	0
P-SSM2	252,407	329	331	300	0	21	7	9	1	1

*GenBank predictions contain multiple overlapping hypothetical proteins at the same locus.

### Conclusions

Despite the wide recognition of the importance of phage, the field of marine phage genomics, as well as phage biology in general, have suffered from technical limitations including (i) limited prior knowledge of the target genome sequence and (ii) restrictive quantities of template DNAs. Here we successfully address these technological issues by devising and optimizing high-throughput phage DNA extraction from crude phage lysates and library construction protocols for either traditional capillary sequencing or pyrosequencing. We demonstrate the superiority of the pyrosequencing approach to the LASL and WGSL methods. The sequencing advances and automated annotation pipeline described here provide the capacity for high-throughput phage genomics, and hence this study provides a roadmap for using a comparative genomic approach to move the study of phage biology rapidly forward. The availability of complete, annotated phage genomes will provide immediate insight into the functional capabilities of specific phage, hence providing a lens with which to explore the biology of these important organisms. Further, these new phage genomes will also provide an important reference for the interpretation of metagenomic data.

## Methods

### Ethics Statement

N/A

### DNA Preparation Methods

#### From CsCl purified phage preps

Phages were propagated on their *Prochlorococcus* hosts (P-SSP7 on MED4, P-SSM2 on NATL1A, and P-SSM4 on NATL2A) in 2 L volumes and were purified for DNA extraction as described previously [21]. Briefly, cell lysate was incubated for 1 hr with nucleases (RNaseA and DNaseL to final concentrations of 10 µg ml^−1^ and 0.25 SU ml^−1^) to degrade host nucleic acids, 2 M NaCl was then added for a 30 minute incubation, then cell debris was spun out (15,000 rcf, 15 mins., 4°C). Phage particles in the supernatant were precipitated with polyethylene glycol (PEG) 8000 (100 g L^−1^) for 2 h, followed by centrifugtion (15,000 rcf, 15 mins., 4°C) to obtain a PEG-phage pellet. Precipitated phages were purified on a cesium chloride step gradient (steps were ρ = 1.30, 1.40, 1.50, and 1.65) spun at 104,000 rcf for 4 hrs at 4°C in a SW28 swinging bucket rotor, with the visible phage band pulled from between the 1.4 and 1.5 layers. This phage band was dialyzed in Slide-a-lyzer cassettes with a 20 kDa molecular weight cut-off for 30 minutes each against MTM100 buffer (600 mM NaCl, 100 mM TrisHCl (pH = 7.5), 100 mM MgCl_2_) where the NaCl concentration was sequentially decreased with each round of dialysis from 3 M, 1.8 M, and two changes of 600 mM NaCl buffer. DNA was extracted from purified phage particles using the Quantum Prep Plasmid miniprep Kit (BioRad #732-6100) according to the manufacturers instructions, generally yielding <500 ng DNA per 2 L lysate. This DNA was used to construct Linker Amplified Shotgun Libraries (LASL).

#### From phage lysate preps

Extraction of phage DNA directly from crude lysate preps has been well-documented for phage lambda, e.g. references [37]–[39], providing clean DNA in high yield in only 1–2 hrs. Given the potential to use this method for high-throughput DNA extraction, we adapted the method for cyanophage. Using cyanophage lysates prepared as described above, we extracted phage DNA directly by a modification of the method originally described in the Promega Wizard™ Lambda Preps DNA purification system (no longer commercially available): 100 ml of cyanophage lysate was centrifuged (15,000 rcf, 15 min, 10°C) to pellet cell debris which was discarded. The supernatant was decanted gently for further processing. When the nuclease step was used, 40 µl of nuclease mix (0.25 mg/ml RNase A, 0.25 mg/ml DNase I, 150 mM NaCl, 50% glycerol, stored at −20°C) was then added directly to the supernatant and the mixture was incubated at 37°C for 15 min. 4 ml of phage precipitant (33% PEG, 3 M NaCl) was added, followed by incubation on ice for 1 hr, then centrufugation at 10,000 rcf, 10 min, 10°C to pellet phage particles. After gently decanting and discarding the supernatant and draining residual liquid onto a paper towel, the phage pellet was resuspended in 500 µl phage buffer (150 mM NaCl, 40 mM Tris-HCl (pH 7.4), 10 mM MgSO4), and transferred to a 2-ml Eppendorf tube. One ml Purification Resin (product A7181, Promega, Madison WI) was then added and mixed gently by inverting the tube. The resulting slurry was then loaded onto a mini-column (product A7211, Promega, Madision WI) through a 5 ml syringe attached to the column, pushing the slurry through with the syringe plunger. The column was then washed with 2 ml 80% isopropanol, the syringe removed and the minicolumn placed into a 1.5 ml Eppendorf tube and centrifuged (10,000 rcf, 2 min, room temperature) to remove any remaining liquid. Phage DNA was then eluted from the column by adding 100 µl TE Buffer heated to 80°C, then placing the column into a 1.5 ml Eppendorf tube and immediately centrifuging (10,000 rcf, 20 sec, room temperature) to recover the DNA. Phage DNA was stored at 4°C for immediate use or at −80°C for long-term storage. Typical yields using this procedure were 100 ng –1 µg of phage DNA per 100 ml phage lysate, as determined both spectrophotometrically (using a NanoDrop Spectrophotometer) and by estimation vs. a standard on agarose gels. This DNA was used to construct Whole Genome Shotgun Libraries (WGSL) and for 454 sequencing.

### Construction and Sanger Sequencing of Linker-Amplified Shotgun Libraries (LASLs)

To prevent potential contamination with and subsequent amplification of non-specific DNA, strict clean lab procedures were implemented for the sample handling and experimental process steps prior to the linker-mediated whole genome amplification. Where possible, sample manipulations were conducted in an AirClean AC600 PCR workstation (AirClean Systems, Raleigh NC) with a dedicated set of pipettes. Tubes, tube caps and all reagents with the exception of DNA, oligodeoxynucleotides, dNTPs, enzymes, SYBR Green I Nucleic Acid Gel Stain and agarose were subjected to UV treatment for 10 min in a Stratalinker UV crosslinker (Stratagene, La Jolla CA, Model 1800). Primer and linker solutions were prepared using UV-treated water, buffers and/or salt solutions. A policy of single-use aliquots for all reagents was adopted. In addition, the gel electrophoresis equipment dedicated for the first size fractionation step was decontaminated with diluted bleach solution (10∶1) prior to use.

LASL libraries were prepared as outlined in Figure 1. Cyanophage gDNA or T7 DNA (Epicentre, Madison WI) samples of 0.5 to 100 ng were adjusted to a final volume of 500 µl by the addition of 10 mM Tris-HCl, pH 7.5, 0.1 mM Na_2_EDTA, transferred into disposable borosilicate glass tubes with polypropylen screw caps (Fisher Scientific, Pittsburgh PA) and sheared using the S2 Adaptive Focused Acoustics (AFA) Instrument (Covaris Inc., Woburn MA). Shearing conditions were as follows: Time  = 35 sec, duty cycle  = 5, intensity  = 5, cycles per burst  = 200, bath temperature  = 6–8°C (chiller set to 4°C). using the End-iT DNA End-Repair Kit (Epicentre, Madison WI). The sheared DNA sample was concentrated (to ≤35 µl) via ultrafiltration using a Microcon YM-100 centrifugal filter unit (Millipore, Billerica MA) following exactly the manufacturer's guidelines. Using the End-iT DNA End-Repair Kit (Epicentre, Madison WI), sheared DNA fragments were incubated in a 75 µl reaction volume (50 µl DNA sample, 8 µl 10X End-Repair Buffer, 8 µl dNTP Mix (2.5 mM each), 8 µl 10 mM ATP, 2 µl nuclease-free water, 4 µl End-Repair Enzyme Mix) for 45 min at 25°C. Reaction clean-up was performed via a MinElute spin column (QIAGEN, Valencia CA). The purified, end-repaired DNA fragments were eluted from the column with 33 µl 10 mM Tris-HCl, pH 8.0. Subsequently, 75 pmol of hemi-phosphorylated linker-A (Figure S2) were ligated to the purified, end-repaired DNA fragments (≤30 µl) in 50 µl containing 10 U Fast-Link DNA Ligase and 0.5 mM ATP (Fast-Link DNA Ligation Kit, Epicentre, Madison WI) for 2 hrs at 23°C. This provided the binding site for the phosphorylated primer PCR-A. Excess linker was removed by subsequent reaction clean-up via a MinElute spin column (QIAGEN, Valencia CA) according to the manufacturer's instructions. The purified, linker-ligated DNA fragments were eluted with 22 µl 10 mM Tris-HCl, pH 8.0. To avoid preferential amplification of very small DNA fragments and to maximize the yield of PCR product in the desired size range, the linker-ligated DNA fragments were subjected to preparative gel electrophoresis: The sample was supplemented with Blue/Orange Loading Dye (Promega, Madison WI) and loaded into two adjacent wells of a 1% SeaKem GTG Agarose (Cambrex Bio Science Rockland, Inc., Rockland MA) gel in 1X Tris-acetate-EDTA (TAE) buffer. After loading 60 ng of a 1 kb DNA Ladder (Invitrogen, Carlsbad CA) on either side of the sample, electrophoresis was performed for 2 hrs at 3.5 V/cm (gel size  = 12 cm width ×14 cm length, gel volume  = 80 ml, well size  = 7.2 mm width, 1.5 mm thickness). Marker lanes (100 ng 1 kb DNA Ladder (Invitrogen, were stained for 30 min with SYBR Green I Nucleic Acid Stain (Invitrogen, Carlsbad CA). The gel slice containing the 1.25–1.75 kb fraction of the sample was excised from the unstained preparative portion of the gel, and the DNA was recovered using the MinElute Gel Extraction Kit (QIAGEN, Valencia CA). The final eluate volume was ∼30 µl. Prior to the large-scale amplification of the linker-ligated, size-fractionated DNA fragments, a PCR titration experiment was performed to determine the lowest possible number of amplification rounds suitable to yield sufficient DNA material in the desired size range for the subsequent process steps, thereby keeping the error rate as low as possible, and to prevent amplification of any co-purified DNA fragments smaller or larger than 1.25–1.75 kb. Four 25 µl PCRs were assembled, each consisting of 1.4 µl linker-A-ligated, size-fractionated DNA fragments, 12.5 µl PfuTurbo Hotstart 2X Master Mix (0.1 U/µl; Stratagene, La Jolla CA), 0.5 µl (5 pmol) phosphorylated primer PCR-A (Figure S2), and 10.6 µl nuclease-free PCR-grade water. Thermocycling included an initial denaturation step (95°C, 2 min), followed by 18, 22, 25 or 28 cycles, respectively, at 95°C (30 sec), 60°C (1 min), and 72°C (1.5 min), and ended with an additional extension step at 72°C for 10 min. PCR products were analyzed via gel electrophoresis. For the large-scale amplification step, a total of seven 25 µl PCRs were performed using exactly the same conditions but the optimal cycle number, as determined in the initial titration experiment. The phosphorylated PCR products were purified with the QIAquick PCR Purification Kit (QIAGEN, Valencia CA), quantitated via PicoGreen fluorescence (Quant-iT dsDNA High Sensitivity Assay Kit, Invitrogen, Carlsbad CA), and subsequently incubated in the presence of a 300-fold molar excess of *Bst*XI/*Eco*RI adaptor (Invitrogen, Carlsbad CA), 10 U Fast-Link DNA Ligase and 0.5 mM ATP (Fast-Link DNA Ligation Kit, Epicentre, Madison WI) in a reaction volume of 50 µl for 2 hrs at 23°C. Excess adaptor molecules were removed by reaction clean-up (QIAquick PCR Purification Kit, QIAGEN, Valencia CA), followed by preparative gel electrophoresis (1.5 V/cm, 18 h) on a 1% SeaKem GTG Agarose gel in 1X TAE buffer (gel size  = 23 cm width ×40 cm length, gel volume  = 600 ml, well size  = 7.2 mm width, 1.5 mm thickness). The DNA samples were divided into two to four wells, and 100 ng 1 kb DNA Ladder (Invitrogen, Carlsbad CA) was loaded on either side of the sample. After electrophoresis, Marker lanes were stained as described above. DNA fragments in the 1.25–1.75 kb size range were eluted using the QIAquick Gel Extraction Kit (Invitrogen, Carlsbad CA). A 10 ng aliquot of the gel-purified *Bst*XI/*Eco*RI linker-ligated PCR products was ligated with 7.5 ng *Bst*XI-linearized low-copy-number cloning vector in the presence of 5 U Fast-Link DNA Ligase and 1 mM ATP (Fast-Link DNA Ligation Kit, Epicentre, Madison WI) in a volume of 25 µl for 2 hrs at 23°C. After heat-inactivation of the ligase in the presence of 0.3 M NaCl for 10 min at 65°C, the reaction buffer was exchanged via ultrafiltration through a Microcon YM-100 centrifugal filter unit (Millipore, Billerica MA) by washing and retrieving the retentate with 10 mM Tris-HCl, pH 7.5, 0.1 mM Na_2_EDTA. 1 µl of the purified ligation products was transformed by electroporation of *Escherichia coli* DH10B (ElectroMAX DH10B-T1^R^ Electrocompetent Cells, Invitrogen, Carlsbad CA). Transformants were selected on Luria-Bertani (LB) agar plates containing 25 mg/ml chloramphenicol and 5% sucrose. Plasmid DNA was prepared by standard protocols, and cloned inserts were bidirectionally sequenced with M13 forward and M13 reverse primers using the BigDye Terminator v3.1 chemistry (Applied Biosystems, Foster City CA). Sequencing reactions were analyzed on ABI3730xI capillary electrophoresis sequencers (Applied Biosystems, Foster City CA).

### Construction and Sanger Sequencing of Standard Whole Genome Shotgun (WGSL) Libraries

WGSL libraries were prepared as outlined in Figure 1. Shearing of 50 to 1000 ng cyanophage gDNA or T7 DNA (Epicentre, Madison WI) was performed using the Covaris AFA technology exactly as described for the construction of LASLs. Sample concentration and simultaneous removal of sheared DNA fragments of low molecular weight (≤200–250 bp) was achieved using the AMPure Kit (Agencourt Bioscience Corporation, Beverly MA), following exactly the manufacturer's recommendations, with a sample to bead ratio of 1 to 0.8 (i.e. 400 µl AMPure reagent added to 500 µl sheared DNA). DNA fragments (>250 bp) were eluted in 54 µl 10 mM Tris-HCL, pH 8.0. Using the End-iT DNA End-Repair Kit (Epicentre, Madison WI), the sheared and concentrated DNA fragments were converted to blunt-end, 5′-phosphorylated DNA fragments in a volume of 80 µl (50 µl DNA sample, 8 µl 10x End-Repair Buffer, 8 µl dNTP Mix (2.5 mM each), 8 µl ATP, 2 µl nuclease-free water, 4 µl End-Repair Enzyme Mix) for 45 min at 25°C. After a purification step using the MinElute Reaction Cleanup Kit (QIAGEN, Valencia CA) and quantitation via PicoGreen fluorescence (Invitrogen, Carlsbad CA), the end-repaired fragments (28 µl) were ligated to *Bst*XI/*Eco*RI adaptor molecules (molar ratio of linker to fragment  = 300 to 1, calculated for 200 bp, the smallest fragment size) using Epicentre Fast-Link DNA Ligation Kit (see construction of LASLs). Excess adaptor was removed by two subsequent purifications via MinElute spin columns (QIAGEN, Valencia CA), followed by preparative gel electrophoresis exactly as described for the second size fractionation step in the the LASL protocol. After staining the Marker lanes with SYBR Green I Nucleic Acid Gel Stain (Invitrogen, Carlsbad CA), the 1.25–1.75 kb DNA fragments were recovered from the unstained preparative part of the gel using the QIAquick Gel Extraction Kit (Invitrogen, Carlsbad CA). Half of the eluted linker-ligated DNA fragments (13 µl) was incubated in the presence of 5 ng *Bst*XI-linearized low-copy-number cloning vector, 4 U Fast-Link DNA Ligase and 1 mM ATP (Fast-Link DNA Ligation Kit, Epicentre, Madison WI) in a 20 µl volume for 2 hrs at 23°C. After heat-inactivation and desalting of the reaction (see construction of LASLs), the ligation products were cloned via electroporation into *Escherichia coli* DH10B (Invitrogen, Carlsbad CA). Paired-end reads from cloned plasmid inserts were sequenced as described for LASLs.

### Construction and Pyrosequencing of 454 Libraries

454 libraries were prepared as outlined in Figure 1. Shearing of 1 to 1000 ng of cyanophage gDNA generated from by either CsCl or Lysate preparations, or T7 DNA (Epicentre, Madison WI) was performed using the Covaris AFA technology and the following conditions: time  = 240 sec, duty cycle  = 5, intensity  = 5; cycles per burst  = 200, and temperature  = 3°C. To concentrate the DNA and to remove sheared fragments below 200 bp, 0.8x the volume (80 µl) of AMPure PCR purification beads (Agencourt Bioscience Corporation, Beverly MA) were added to the 100 µl sheared volume and vortexed for 30 sec. The beads with the captured DNA were immobilized using a Dynal MPC-S magnet until the solution was clear. The supernatant was discarded, and 200 µl of 70% ethanol was added and incubated for 30 sec. Using a Dynal MPC-S magnet, the ethanol was removed and the beads were dried at room temp to remove any residual ethanol. The DNA shearing profile was determined by running 1 µl of the samples on the Agilent Bioanalyzer 2100 using a DNA 1000 chip (Agilent Technologies).

All sample prep reagents were provided in the GS20 or FLX Library Preparation Kit (454 Life Sciences, Branford CT), and the process was performed according to Margulies et al.[33] with slight modifications as follows: fragment end polishing, adaptor ligation, and library immobilization reactions were carried out as described except for the clean-up steps, which were performed with the addition of 1.8x AMPure beads as above described. Finally, the single strand template was melted off the beads using the Dynal MPC-S magnet with 25 µl of 250 mM sodium hydroxide. The supernatant containing the single stranded template was transferred to a new tube and another 25 µl of 250 mM sodium hydroxide. This denaturation step was repeated, and the two 25 µl aliquots of single strand template were pooled. The denatured aliquots (50 µl total) were neutralized with 1.24 µl of 10% acetic acid and concentrated with 1.8x AMPure beads (90 µl) as described above. The final elution volume of the single stranded template was 25 µl using the EB buffer (QIAGEN, Valencia CA). The single strand DNA profile and quantification was determined by running 1 µl of the samples on the Agilent Bioanalyzer 2100 using a RNA Pico 6000 chip. The concentration (pg/µl) was then used to calculate the number of molecules/µl of the final product: [single strand DNA (pg/µl)]/[MW of nucleotide (325) x base pair length of DNA strand] x [6.02×10^23^]. The single-strand templates were quantified using the Pico 600 Chip (Agilent, Santa Clara CA) and were diluted to a normalized concentration of 1×10^8^ molecules/µl for the emulsion PCR reactions. The single stranded DNA material recovered by from 1 ug and 100 ng could be detected and quantified on an Agilent Pico 6000 chip. The amount of single stranded material recovered from the 100 ng starting material was 10x less than that recovered from the 1 ug of starting DNA. Therefore, an estimate of 100x and 1000x less material was calculated for single stranded DNA recovered from the 10 ng and 1 ng starting amounts, respectively, to estimate the appropriate dilution necessary to obtain 1×10^8^ molecules/µl. Emulsion PCR and sequencing was performed without modifications according to the GS20 protocol. Emulsion PCR and sequencing was performed without modifications, according to the GS20 of FLX protocol.

### Comparative Quantitative PCR Analysis

The relative representation of 12 P-SS2 specific genomic regions following Covaris shearing, LASL and WGSL treatments was determined by comparison to purified gDNA using real-time quantitative PCR. Primer sets were designed using the Primer3 software package (http://frodo.wi.mit.edu/cgi-bin/primer3/primer3_www.cgi) and chosen to span regions of the genome that had both high and low sequence coverage as indicated by assembly results (Figure S1). qPCR reactions were performed in duplicate, consisting of 1 µl DNA template (0.5 ug total), 2 µl each of forward and reverse PCR primers (final concentration of each primer: 100 nM), 10 µl 2X Brilliant SYBR Green qPCR Master Mix (Stratagene, La Jolla CA), 0.3 µl diluted reference dye (Stratagene, La Jolla CA), and 4.7 µl nuclease-free PCR-grade water. Reactions were initiated with 10 min of incubation at 95°C, followed by 40 cycles of 95°C (30 sec), 60°C (1 min), and 72°C (1 min) using the Mx3005P qPCR System (Stratagene, La Jolla CA). Data analysis was performed by using the MxPro qPCR software package (Stratagene, La Jolla CA).

Applying the efficiency-corrected comparative quantitation method [40], results were calculated as “relative quantity to the calibrator,” where the calibrator sample (purified P-SS2 gDNA) is assigned an arbitrary quantity of “1” and the unknown samples (LASL PCR intermediate and WGSL ligation) are expressed in terms of their fold difference (variation) to this sample. One assay (Amplicon identifier 98) served as normalizer for all other assays.

### Genome Assembly

Phage genomes were assembled using either the ARACHNE [41] or Newbler (454 Life Sciences, Branford CT) assembly software packages. We assembled sequence data generated by the WGSL and LASL methods using the Assemblez module of ARACHNE with all the default settings except ‘recycle_bad_contigs’ which was set to ‘false’ to ensure the assembler did not exclude small, low-coverage contigs. We assembled data generated by 454 using the Newbler software package with all settings set to default and the ‘-finish’ mode invoked. The ‘-finish’ mode will create sequences through repetitive regions that form unambiguous paths between contigs. With this mode active some regions that would typically generate a gap in the assembly due to repetitive sequence are assembled. Resulting contigs from both assembly methods were manually inspected for quality and joined as appropriate using an in-house sequence editor. Comparisons of 454 assemblies to available reference sequences were performed using MUMmer v3.20 [42] run with default options. Variations between the two sequences where called using the *show-snps* utility.

We evaluated the minimal sequencing coverage required to obtain a full-length, high-quality genome using *in silico* assembly experiments. Sequence reads from a single genome (Cyanophage S-SM1) were randomly parsed to achieve sequences coverages ranging from approximately 5x to 30x. These read sets where then assembled using the Newbler approach just described.

In addition we evaluated assembly performance using *in silico* experiments for instances when multiple phage are present in a single sample. Isolating individual cyanophage is sometimes difficult and occasionally multiple types of cyanophage are present in samples. We therefore explored whether sequencing mixed samples could potentially yield complete, assembled cyanophage genomes. To simulate the assembly of mixed samples in a sequencing run, we used randomly fragmented subsets of four cyanophages genomes: P-SSM4, P-SSP7, P-SSM2, MED4–247, and MED4–259, comprising both myo- and podoviruses with sizes of approximately 50 kb to 252 kb (Table S2), and attempted to reassemble them using Newbler.

To simulate sequencing and assembly, we defined the following parameters for each simulated run (Figure S3).

- *g*: the number of genomes in the sample, ranging from 1–5
- *n*: the bp size of the randomly generated fragments for each genome
- *(i,j)*: the variability in length of each fragment
- *x*: the number of reads per run, set to 600,000
- *ratio*: the ratio of sequences from each read

After the parameters were set, randomly generated FASTA-formatted fragments totaling *x*, the number of reads per run, were created from each of *g* genomes. These fragments were submitted to Newbler for assembly as previously described. For these experiments a “successful assembly” was determined as follows: each long contig from the Newbler output file “454LargeContigs.fna” was input to the genome alignment program MUMmer v3.20 [42] for whole-genome alignment with the set of five selected cyanophage (Table S2). MUMmer finds the maximal exact matches between two input sequences [42]. Contigs with greater than 98% aligned sequence to a target cyanophage were considered assembled.

### Genome Annotation

Full-genome phage assemblies were annotated using a semi-automated workflow that: (i) identifies putative open reading frames (ORFs) using evidence-based approaches and *ab initio* gene prediction and (ii) selects genes based on evidence and a set of rules. Specifics of these processes are as follows:

#### Evidence-based ORF identification

BLAST and Pfam evidences are central to the whole gene annotation process. A set of raw alignments is produced by blast (blastx) homology search [43] of the whole genome against Genbank's non-redundant protein database (NR). Individual blast alignments are clustered by linking neighboring alignments derived from the same protein in NR. A set of overlapping blast clusters on the genomic region represents a blast locus on the genome assembly. Blast hits with e-values less than 1e-10 are used as blast evidence. HMMER [44] searches are run against the Pfam [45] and TIGRfam [46] libraries to find protein domains. Ribosomal RNAs (rRNAs) are identified with RNAmmer [47]. The tRNA features are identified using tRNAScan [48]. Other common RNA features are identified with Rfam [49] on six-frame translations of the genomic sequence (e<0.01).

#### Gene Model Prediction

The gene caller for protein coding genes uses *ab initio* and evidence based gene predictions. *Ab initio* gene models are predicted using the computational gene prediction programs: GeneMarkS [50], Glimmer3 [51], MetaGene [52], Zcurve [53], GISMO [54], and Genewise [55]. Default settings are used for all with the exception of Glimmer3 where a minimum length of 90 bp and genetic code table 11 are used.

Blast [43] evidence-based genes are predicted by an in-house application *findBlastOrfs*. It uses blastx alignments to build a complete gene model from the hits, and is particularly useful in low-coverage genomes with low quality regions, frame shifts or gaps, where a*b initio* gene predictors generally predict no or incorrect gene models. The *findBlastOrfs* program can successfully predict single genes that are disrupted by gaps and/or frameshifts where other prediction methods create truncated or split genes.

When gene models from well-annotated reference genomes are available, they are transferred to the intended genome assembly to improve the annotation process. This method is a two-step procedure. First, it finds collinear (synteny) blocks between the two genomes by creating pair-wise alignments and then it generates a global alignment for the entire region that covers the collinear blocks. In the second step, a gene mapping program is used to transfer annotations from the reference to the target genome within the specific syntenic blocks.

Gene models are manually checked for errors such as in-frame stops, very short proteins, splits, merges, etc. When two or more loci are merged by the gene caller which can be deduced from the blast and Pfam evidence, they are manually annotated as separate gene models. Similarly, gene models split by the gene caller are manually merged as a single locus as appropriate. If blast loci are missing (false negatives) by the gene caller, new annotations are made and false positives are removed.

#### Consensus gene model selection

Identification of protein-coding genes was performed with the Broad Institute's Calhoun annotation infrastructure, a set of algorithms that uses a rule-based selection process to evaluate the evidences and build consensus gene models. The above *ab initio* and blast evidence based gene models and manual gene models are clustered into potential gene loci. The most likely non-conflicting gene models are selected at a given locus, based on Pfam evidence and protein length agreement with the BLAST hits. Genes with overlap to non-coding RNA and other loci are also checked. Questionable gene models are tagged appropriately with curation flags and notes. This information is used for manual annotation and quality checking. Manual annotators resolve splits, merges, and overlaps and refine the gene product names when possible.

## Supporting Information

Figure S1 Fold variation in amplification of amplicons spanning high and low coverage regions in the P-SS2 genome as determined by qPCR following Covaris shearing (A), Linker Amplificaiton Shotgun Library construction (B), and Whole Genome Shotgun library construction (C). Variation is relative to unprocessed gDNA and results across the amplicons are normalized using primer pair 98. Multiple starting template DNA quanities were assayed for LASLs. Average sequence coverage in P-SS2 genome across qPCR amplicons (D).
(5.82 MB TIF)

Figure S2 Detailed description of LASL linkers, primers, and constructs.
(5.82 MB TIF)

Figure S3 Overview of mixed sample in silico assembly analysis.
(0.81 MB TIF)

Table S1 Detailed comparison of 454 phage assemblies to existing NCBI RefSeqs.
(0.05 MB PDF)

Table S2 Phage genomes included in in silico mixed sample experiments.
(0.04 MB PDF)
