# Supplementary figures and images for: Analysis of High-Throughput Sequencing and Annotation Strategies for Phage Genomes
Source: PLoS One. 2010 Feb 5;5(2):e9083. doi: 10.1371/journal.pone.0009083 (PMC2816706; doi:10.1371/journal.pone.0009083)

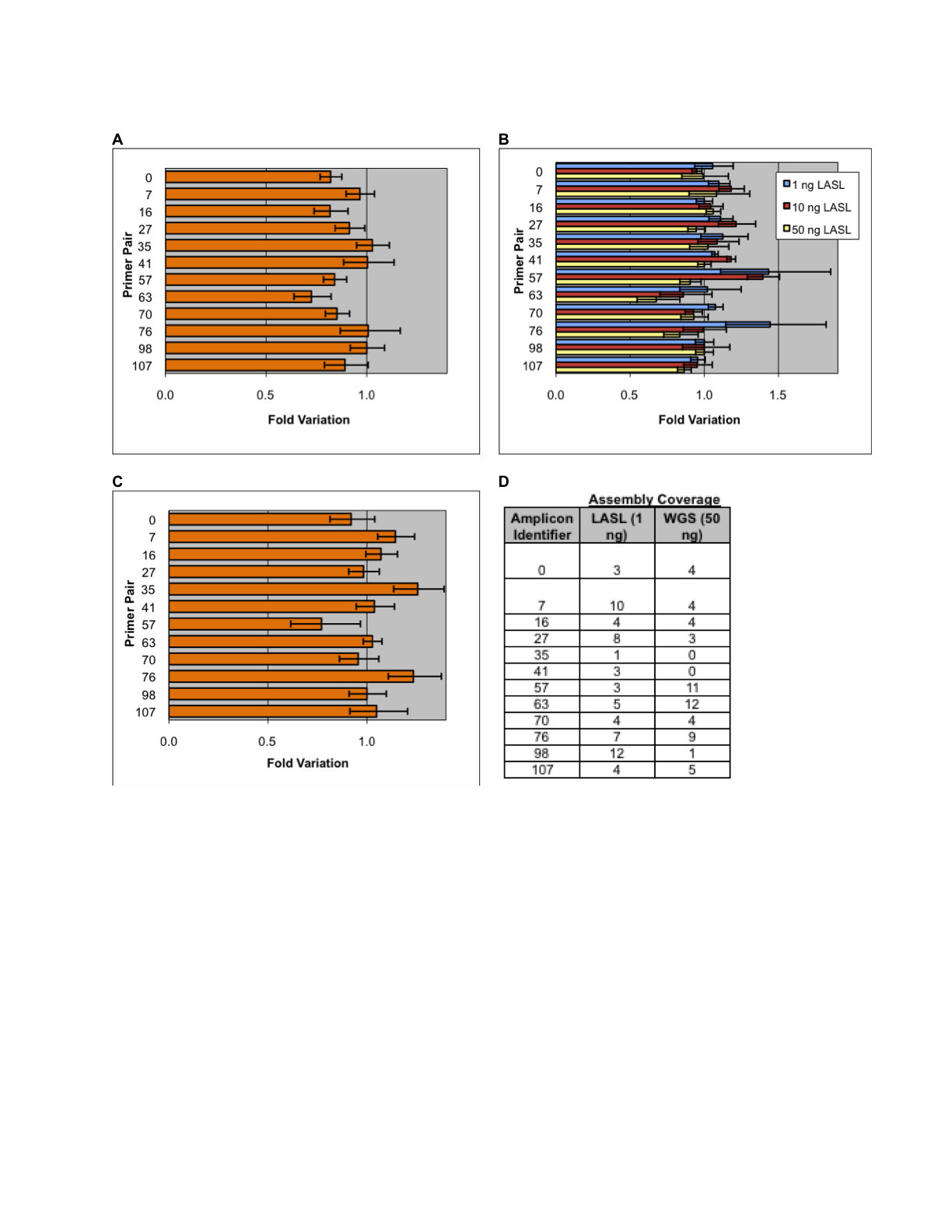

Supplement: Figure S1 — Fold variation in amplification of amplicons spanning high and low coverage regions in the P-SS2 genome as determined by qPCR following Covaris shearing (A), Linker Amplificaiton Shotgun Library construction (B), and Whole Genome Shotgun library construction (C). Variation is relative to unprocessed gDNA and results across the amplicons are normalized using primer pair 98. Multiple starting template DNA quanities were assayed for LASLs. Average sequence coverage in P-SS2 genome across qPCR amplicons (D). (5.82 MB TIF) [file pone.0009083.s001.tif]

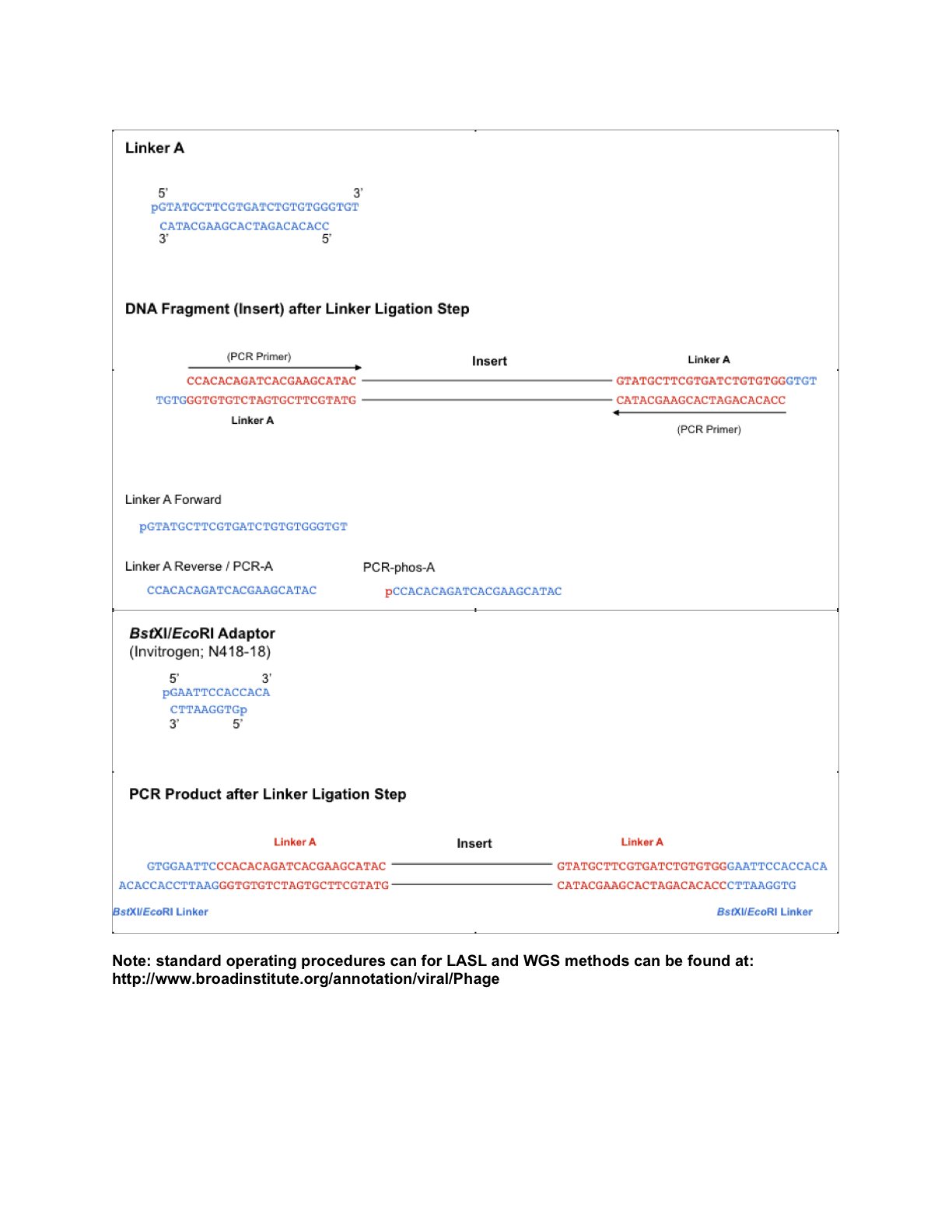

Supplement: Figure S2 — Detailed description of LASL linkers, primers, and constructs. (5.82 MB TIF) [file pone.0009083.s002.tif]

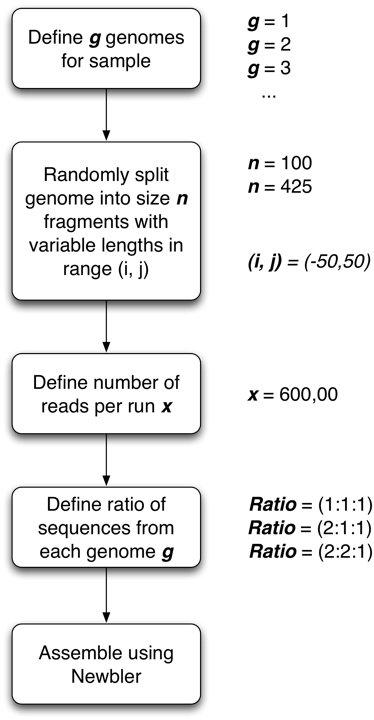

Supplement: Figure S3 — Overview of mixed sample in silico assembly analysis. (0.81 MB TIF) [file pone.0009083.s003.tif]
