# Supplementary material for: Analysis of High-Throughput Sequencing and Annotation Strategies for Phage Genomes
Source: PLoS One. 2010 Feb 5;5(2):e9083. doi: 10.1371/journal.pone.0009083 (PMC2816706; doi:10.1371/journal.pone.0009083)
Supplement: Table S1 — Detailed comparison of 454 phage assemblies to existing NCBI RefSeqs. (0.05 MB PDF) [file pone.0009083.s004.pdf]

Supplementary Table 1. Detailed comparison of assembled 454 phage assemblies to existing NCBI RefSeqs

P-SS2

Event ID	Event Type	Reference Position	Reference Base	Assembly Base
1	Substitution	25	A	T
2	Substitution	26	A	C
3	Substitution	27	T	A
4	Insertion	50	.	C
5	Substitution	51	G	A
6	Substitution	52	C	G
7	Insertion	5686	.	T
8	Deletion	9867 - 10518 (652bases)	*	.
9	Deletion	10519 - 11170 (652 bases)	*	.
10	Insertion	12963	.	A
11	Insertion	20387	.	A
12	Insertion	24818	.	A
13	Insertion	25770	.	T
14	Insertion	26323	.	A
15	Insertion	27909	.	T
16	Insertion	29796	.	T
17	Insertion	72840	.	A
18	Insertion	83362	.	T
19	Insertion	84191	.	T
20	Deletion	90834	G	.
21	Insertion	92064	.	T
22	Insertion	92170	.	A
23	Deletion	101663 - 102460 (707 bases)	#	.
24	Substitution	107450	C	T
25	Substitution	107451	A	G
26	Substitution	107452	G	A

* cctatgagcatcttcttgctggtgtttacgaccgtaagaaggaggacgtttcttttgagttcttgttatctcgttatgacagagccgacctagaactcgaggcagctgatggggcttgcactagt
gctgcatgcctggccaaagctgagaagttagagcgtgagggagtctcgagtgtagaatcaaccgatcagcataagaaggattagcggtagattgacataaggacatggccacgcca
tgatctgggagggttaagccctccctttcctttgacccttacagcttttcaaatgactgattcacgattgaggttgctggcaatggccaccttgaccttgacggtttctggggcagctatggcat
tagctaggcaacccatccctcaggttcctgcagccgctgccagcgcaccattaacacctccacccccagttccacagcacgtagccctcaagcccttagcggaccttgtgggtgatgta
gagagcaaatcagcaggcggatataacgcagcaaacaatggctatgccatgggcttaggtagccatggacttagtaagcacttcggtactgactgtaagaacatcagcgttggccag
atcatcaaggcccaacagcgtggctggctttatgcagttggtcgctacc

\# gctagtcaccaagtcgaacaagaactggtgccaggcatacctaccgcaactacgtcgagaagtcattgtccatgacgctcgaaacatctacgaactacccaaatccgaaaaatgact
gagcaacaactcgaaagccttcagcaacaagccacccgtcttctgactgagtcgatggagatccagcgaatgatcagcgcatgcatcgaagatcagcgccgcattgctcacaagta
caacaacgacgttccttttgagctggtcgaggaattccatcctgatgaggagctttacgcacaagtccttgcagcatctaagagggggcagctgtgatcgaagttcgccttgacatgcctc
ttatgtccaaggctcgcccacgggtggccaggggcagggcttacatgcctgctccttatgtgaattggaaaaagaccgcacaggtacaactcgagtccttgtggactgaaaacgatctc
ccgacatatgacaaggagacgcctattaagctcagtgttcaggctcatggtcctggtcgttgtgatagcgacaacctcttaggtgctctgctcgatgccggcctgcccgttaagcagggtt
ggcgtggttgttgggtcgacgaccgagtgacagttatcccatgggtctgctttgaatgggtgaaaaactctgagcaattctgggtacttcagatcaatcaggtttagttgatatagctcattttt
ggtatagccctataccaatatctcgcctttacctgagaggcttctgagatttgaccaagagttgacacttcgcccttga

P-SS2 Summary

There are 14 single nucleotide Insertions and 4 Deletions. 2 of these deletions are 652bp regions and 1 is a 707bp region representing tandem repeats collapsed by Newbler.

The P-SS2 genomes have a sequence identity of 99.39% (% identity is calculated from aligned bases only)

P-SSM2

Event ID	Event Type	Reference Position	Reference Base	Assembly Base
1	Substitution	4099	A	C
2	Substitution	4268	G	A
3	Substitution	54287	G	A
4	Substitution	54405	C	T
5	Substitution	69999	G	A
6	Deletion	71091	C	.
7	Substitution	179607	G	A
8	Insertion	229811	.	A,T,T,A,C,A,T
9	Substitution	236044	G	A

P-SSM2 Summary

In the P-SSM2 454 assembly, there are 7 Substitution events, 1 Deletion and 1 seven base Insertion event consisting of the 7 nucleotides listed in SNP Event ID 8.

The P-SSM2 genomes have a sequence identity of 99.99% (% identity is calculated from aligned bases only)

P-SSP7

Event ID	Event Type	Reference Position	Reference Base	Assembly Base
1	Insertion	1540	.	T
2	Insertion	1542	.	T
3	Gap in assembly	2697 - 2739 (43bases)	#	.
5	Insertion	8559	.	A
6	Insertion	19756	.	T
7	Insertion	21054	.	T
8	Insertion	29724	.	A
9	Substitution	35157	T	G
10	Substitution	37197	G	A
11	Insertion	38119	.	A
12	Insertion	38571	.	A

CCATGAAGACGTAGCGTGGAAAGCCAAGGACTGGTGTGATACC

P-SSP7 Summary

In the P-SSP7 454 assembly, there are 8 Insertions and 2 Substitutions. The P-SSP7 454 assembly is in three contigs. Event-3 shows Sanger draft reference sequence which is lost in the 454 assembly when the contigs failed to join and created a gap. There are 454 reads that mapped to these regions in Sanger reference but did not assemble

The P-SSP7 genomes have a sequence identity of 99.46% (% identity is calculated from aligned bases only)

T7

Event ID	Event Type	Reference Position	Reference Base	Assembly Base
1	Substitution	1114	C	A
2	Insertion	1895	.	A
3	Substitution	24554	A	G
4	Substitution	27212	A	G
5	Substitution	34976	C	A

Note: NCBI T7 RefSeq contains a 160 base-pair terminal repetition that is not part of the T7 strain sequenced
TCTCACAGTGTACGGACCTAAAGTTCCCCCATAGGGGGTACCTAAAGCCCAGCCAATCACCTAAAGTCAACCTTCGGTTGACCTTGAGGGTTC
CCTAAGGGTTGGGGATGACCCTTGGGTTTGTCTTTGGGTGTTACCTTGAGTGTCTCTCTGTGTCCCT

T7 Summary

In the T7 454 assembly, there are 4 Substitution and 1 Insertion events.

The T7 genomes have a sequence identity of 99.99% (% identity is calculated from aligned bases only)
