# Supplementary material for: Analysis of High-Throughput Sequencing and Annotation Strategies for Phage Genomes
Source: PLoS One. 2010 Feb 5;5(2):e9083. doi: 10.1371/journal.pone.0009083 (PMC2816706; doi:10.1371/journal.pone.0009083)
Supplement: Table S2 — Phage genomes included in in silico mixed sample experiments. (0.04 MB PDF) [file pone.0009083.s005.pdf]

Supplementary Table 2. Phage genomes included in *in silico* mixed sample experiments

Reference Genome	Phage Type	Genome Size (bp)	GC Content (%)	Repeat Content (%)
MED4-259	T4-like myovirus	183,806	38	0.9
MED4-247	T4-like myovirus	181,044	38	1.3
P-SSM4	T4-like myovirus	178,249	37	1.3
P-SSM2	T4-like myovirus	252,401	36	3.3
P-SSP7	Podovirus	44,970	39	1.7
